# Long non-coding RNA *Xist* regulates oocyte loss via suppressing *miR-23b-3p*/*miR-29a-3p* maturation and upregulating STX17 in perinatal mouse ovaries

**DOI:** 10.1038/s41419-021-03831-4

**Published:** 2021-05-25

**Authors:** Meng Zhou, Xiaoqiu Liu, E. Qiukai, Yanxing Shang, Xiaoqian Zhang, Shuting Liu, Xuesen Zhang

**Affiliations:** 1 State Key Laboratory of Reproductive Medicine, Nanjing Medical University, 211166 Nanjing, China; 2 Department of Microbiology, Key Laboratory of Pathogen Biology of Jiangsu Province, Nanjing Medical University, 211166 Nanjing, China

**Keywords:** Autophagy, Oogenesis

## Abstract

The fecundity of female mammals is resolved by the limited size of the primordial follicle (PF) pool formed perinatally. The establishment of PF pool is accompanied by a significant programmed oocyte death. Long non-coding RNAs (lncRNA) are central modulators in regulating cell apoptosis or autophagy in multiple diseases, however, the significance of lncRNAs governing perinatal oocyte loss remains unknown. Here we find that Yin-Yang 1 (YY1) directly binds to the lncRNA X-inactive-specific transcript (*Xist*) promoter and facilitates *Xist* expression in the perinatal mouse ovaries. *Xist* is highly expressed in fetal ovaries and sharply downregulated along with the establishment of PF pool after birth. Gain or loss of function analysis reveals that *Xist* accelerates oocyte autophagy, mainly through binding to *pre-miR-23b* or *pre*-*miR-29a* in the nucleus and preventing the export of *pre-miR-23b*/*pre*-*miR-29a* to the cytoplasm, thus resulting in decreased mature of *miR-23b-3p*/*miR-29a-3p* expression and upregulation *miR-23b-3p*/*miR-29a-3p* co-target, STX17, which is essential for timely control of the degree of oocyte death in prenatal mouse ovaries. Overall, these findings identify *Xist* as a key non-protein factor that can control the biogenesis of *miR-23b-3p*/*miR-29a-3p*, and this YY1-*Xist*-*miR-23b-3p*/*miR-29a-3p*-STX17 regulatory axis is responsible for perinatal oocyte loss through autophagy.

## Introduction

The establishment of the primordial follicle (PF) pool represents the first stage of folliculogenesis and this non-renewable PF resource is crucial for maintaining female reproductive life^1^. As the principal functional reproductive unit that provides mature oocytes, the population of PFs is perinatally established, in a process termed cyst breakdown^2^. Ensuring proper primordial follicle generation and early follicle development is under precise control^1^. However, multiple factors can induce excessive loss or overactivation of PFs in the PF pool, which is closely related to primary premature ovarian insufficiency (POI) and even to female infertility^3,4^. A better understanding of the regulatory mechanisms underlying PF pool formation will provide us with deep insights into the potential pathogenic mechanism of POI^5–7^.

Till now, the main studies on PF pool formation or even the pathogenesis of POI associated with PF formation are restricted to protein-coding genes^8,9^. However, only a small proportion of POI cases can be interpreted by causative genetic defects, such as chromosomal abnormalities and gene mutations^4,8,10,11^. Eukaryotic genomes have a wide range of transcriptional properties, producing a large number of RNA transcripts. Among them, only <2% of these RNAs will serve as mRNA templates, and the remaining part of transcripts are non-coding RNAs (ncRNAs). Obviously, there is a lack of study on the roles of ncRNAs, especially long non-coding RNAs (lncRNAs) at the early stage of folliculogenesis, since these ncRNAs have recently been proved to play essential roles in both physiological and pathological conditions in female reproduction^9^.

LncRNAs are a category of ncRNAs with a length of over 200 nucleotides without protein-coding potential^12^. They can function as a signal, guide, decoy, microRNA sponging, or scaffold to modulate gene expression at transcription and post-transcription levels^13,14^. Several recent studies have linked lncRNAs to the process of folliculogenesis^15–18^, and dysregulation of their expression can cause ovarian diseases, including POI^16,17,19,20^. Besides, transcriptional profiling of lncRNAs in ovarian cortical tissues from women with POI has recently been performed, and 20 differently expressed lncRNAs were identified to be involved in ovarian follicular development^21^. However, whether lncRNAs are participating in the PF pool formation is nearly completely unknown. A recent transcriptome analysis of lncRNA expression in human primordial, primary and small antral follicles was performed and lncRNA X-inactive-specific transcript (*Xist*) is revealed to be highly transcribed throughout the stages tested, suggesting that *Xist* has emerged from the dormant primordial stage of human follicle development^15^. But its biological function and the molecular mechanism underlying the formation of the PF pool have not been clarified.

MicroRNAs (miRNAs) are another class of short ncRNAs with a length of 20–24 nucleotides, which are generated through the initial nuclear cleavage and subsequent cytoplasmic cleavage events^22,23^. The miRNA gene is initially transcribed from the genome into primary transcript (pri-miRNA), followed by cleavage into ~70 nucleotide precursors miRNA (pre-miRNAs). The pre-miRNA is then exported into the cytoplasm where it is further cleaved into an RNA duplex analogous. The mature miRNA is finally generated once one strand is discarded. It is well acknowledged that miRNAs can negatively control the expression of target genes in a wide range of cell signaling pathways related to different physiological and pathological processes^24,25^, including PF formation and even POI^26–29^. However, these studies focus more on how these miRNAs regulate their downstream targets and participate in the regulation of PF formation, and reports of miRNA biogenesis and regulation are missing.

In this study, we showed a time-specific decrease in *Xist* levels from the fetal to neonatal period in the ovaries, which correlates with the upregulated expression of *miR-23b-3p*/*miR-29a-3p*, and a stepwise decrease of STX17. We then demonstrated that *Xist* can block *miR-23b-3p*/*miR-29a-3p* processing, resulting in decreased mature of *miR-23b-3p*/*miR-29a-3p* expression, leading to upregulation of STX17, which is essential for timely control of the degree of oocyte death induced by autophagy in prenatal mouse ovaries.

## Results

### *Xist* is essential for PF pool formation in the perinatal mouse ovaries

**Fig. 1:**
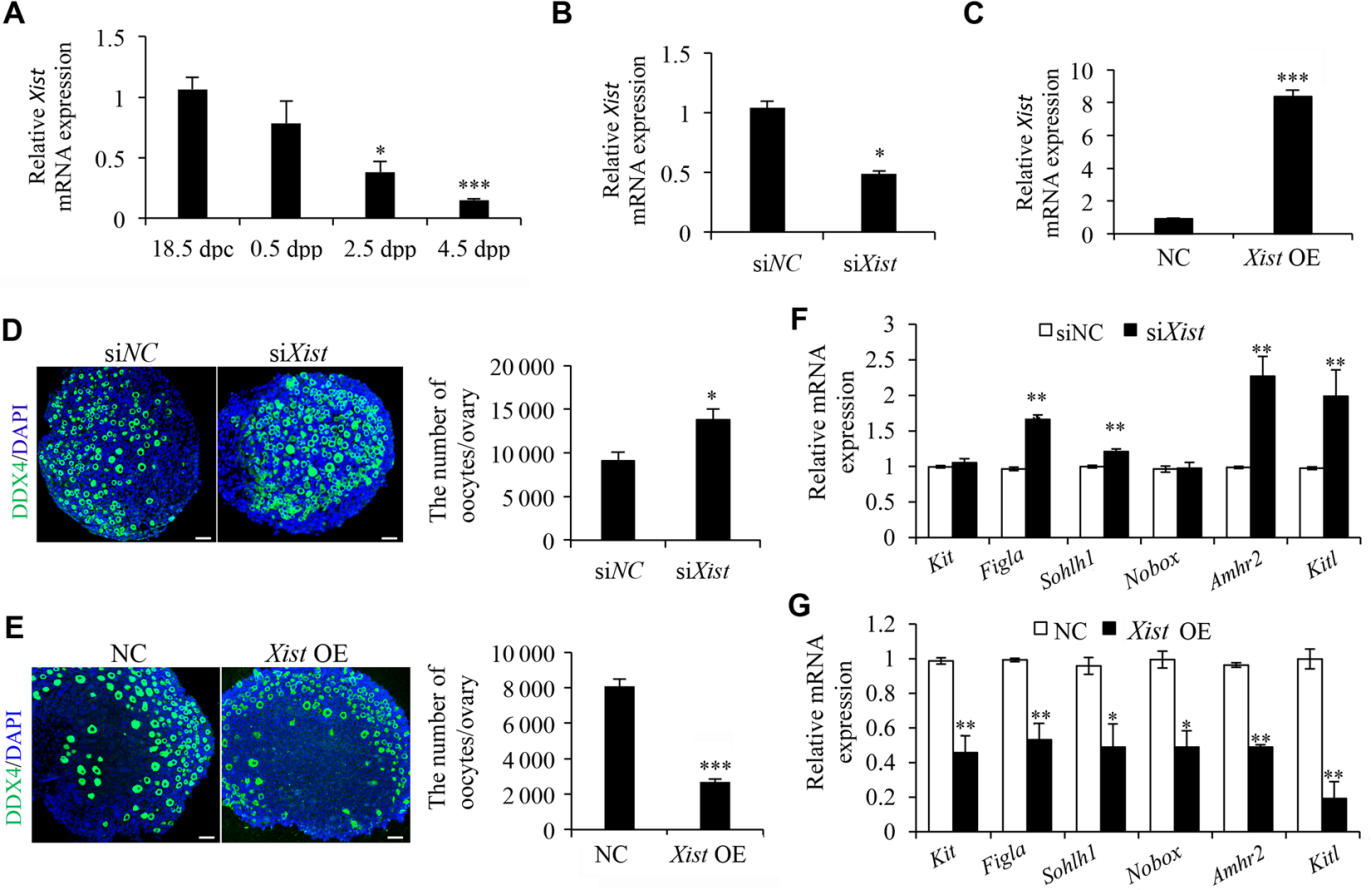
*Xist* expression in perinatal ovaries is essential for PF pool formation. **a** qRT-PCR analysis of *Xist* expression at the indicated time points. *Gapdh* was used to normalize samples. **b**, **c** qRT-PCR analysis of *Xist* expression in newborn ovaries transfected with si*Xist* (**b**), and *Xist* OE (**c**), relative to respective controls. **d**, **e** Representative images of DDX4 immunofluorescence staining (left) and quantification of follicles (right) in newborn ovaries transfected with si*Xist* (**d**), and *Xist* OE (**e**). Scale bars: 50 μm. **f**, **g** qRT-PCR analysis of genes involved in PF survival and development in si*Xist* (**f**), and *Xist* OE (**g**) transfected ovaries, relative to respective controls. Student’s *t*-test: *P < 0.05, **P < 0.01, ***P < 0.001.

To address the physiological significance of *Xist* during the establishment of PF pool in the ovaries of newborn mice, we first showed by qRT-PCR analyses that *Xist* transcripts started to decrease in a time-specific manner from 18.5 d postcoitus (dpc) to 4.5 d postpartum (dpp) (Fig. 1A). This dynamic change of *Xist* just coincides with the timing of massive oocyte loss, indicating that *Xist* may be involved in regulating oocyte loss. To assess this possibility, gain or loss of function experiments were carried out into 0.5 dpp ovaries by separately transfecting with *Xist* siRNA (si*Xist*) and the control siRNA (si*NC*), or *Xist*-pcDNA3.1 (*Xist* OE) and the empty control (NC). The qRT-PCR analysis confirmed that si*Xist* transfection decreased, and *Xist* overexpression increased *Xist* expression in neonatal mouse ovaries after 96 h of in vitro culture (Fig. 1B, C). Subsequent histological analysis showed a significant increase of oocyte number upon *Xist* knockdown compared with the control (Fig. 1D), while *Xist* OE resulted in significantly less available oocytes than did NC (Fig. 1E). In agreement with the changes of oocyte quantification, expression of the genes essential for PF formation also showed an increase in si*Xist*-treated ovaries (Fig. 1F). Alternatively, expression of these genes was inhibited upon *Xist* OE (Fig. 1G). These results indicate that *Xist* may be required for oocyte loss in the newborn ovaries and a decrease of *Xist* is indispensable for PF pool formation.

### *Xist* promotes perinatal oocyte loss mainly through oocyte autophagy

**Fig. 2:**
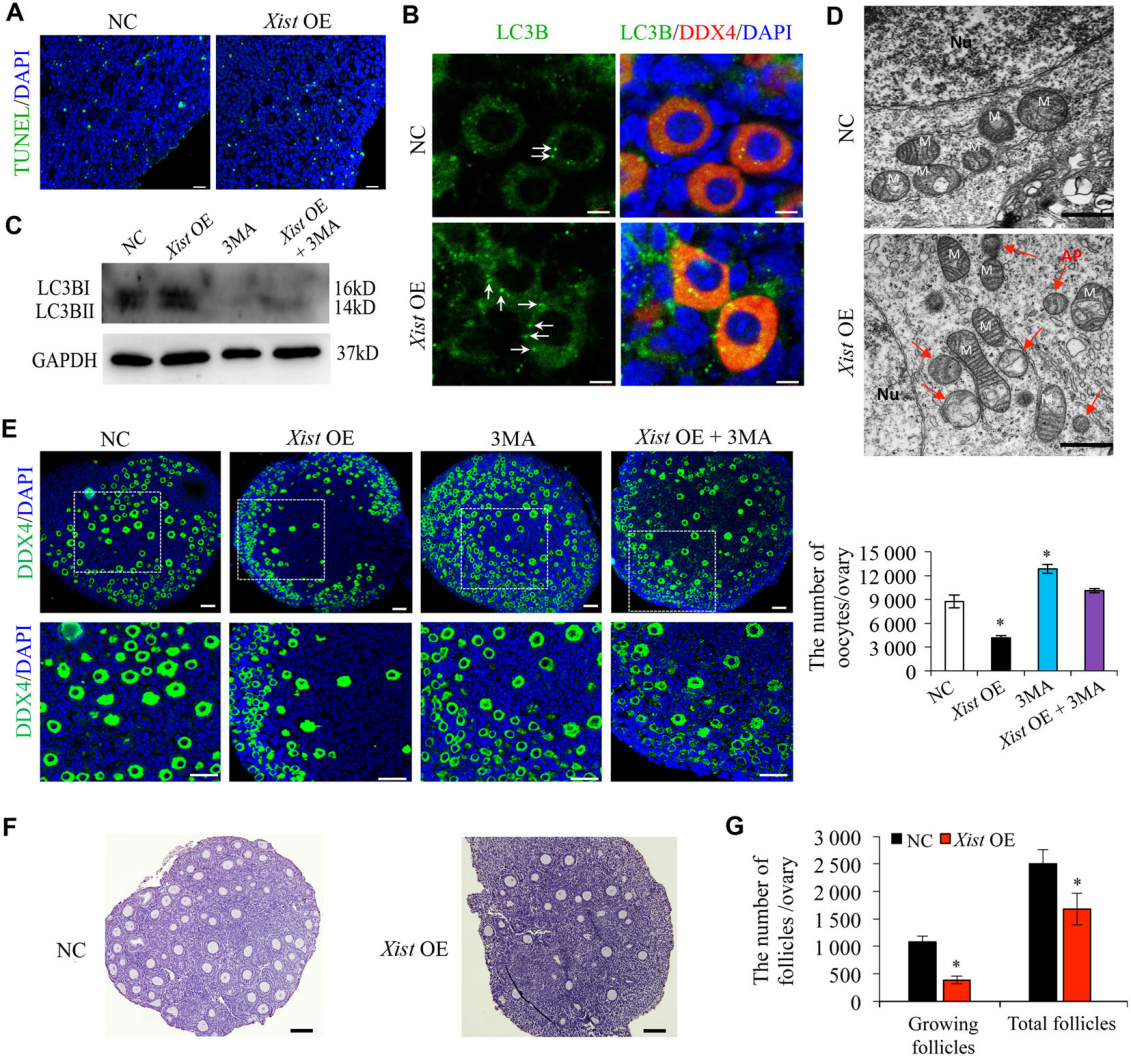
*Xist* promotes oocyte autophagy during PF formation. **a** TUNEL staining in newborn ovaries transfected with *Xist*-pcDNA3.1 (*Xist* OE) or the empty vector control (NC). The nucleus was stained by DAPI (blue). Scale bars: 20 μm. **b** Immunofluorescence staining of LC3B (green), and DDX4 (red) in newborn ovaries transfected with *Xist*-OE compared to NC control. Scale bars: 5 μm. Arrows indicating LC3B puncta. **c** WB analysis of active LC3B expression in newborn ovaries treated under indicated condition. **d** TEM analysis of autophagosomes in newborn ovaries transfected with *Xist*-OE compared to NC control. Red arrow indicating autophagosomes. Scale bars: 1 μm. **e** Representative images of DDX4 immunofluorescence staining (left) and quantification of follicles (right) in newborn ovaries treated under indicated condition. The nucleus was stained by DAPI (blue). Scale bars: 50 μm. **f**, **g** Representative images (**f**) and quantification of follicles (**g**) in the ovary transfected under indicated condition. Scale bar, 20 μm. Student’s *t-*test: *P < 0.05.

It is well acknowledged that apoptosis and autophagy are involved in the process of oocyte death during PF pool establishment^30,31^. To clarify whether *Xist* is involved in the programmed oocyte death through apoptosis and autophagy, we first performed TUNEL analysis to evaluate oocyte apoptosis. Overexpression of *Xist* did not increase the number of TUNEL-positive cells, suggesting that oocyte apoptosis may not be the major cause of oocyte loss upon *Xist* OE (Fig. 2A). As autophagy is also reported to be active during follicular assembly and contributes to perinatal oocyte loss^5^, we, therefore, tested whether *Xist* could induce autophagy in perinatal mouse ovaries. As the microtubule-associated protein light chain 3B (LC3B) is a marker of autophagosomes, we observed more LC3B puncta presented in the cytoplasm of *Xist-*overexpressed oocytes (Fig. 2B). We then incubated the 0.5 dpp ovaries with 3MA, an autophagy inhibitor, and 3MA treatment nearly completely inhibited LC3B protein expression, while *Xist* overexpression could slightly eliminate the negative effect of 3MA on LC3B expression (Fig. 2C). Furthermore, TEM analysis identified the large double-membrane vacuoles filled with degraded organelles in *Xist*-overexpressed oocytes (Fig. 2D), which is the typical appearance of autophagosomes^32^. To further confirm that autophagy is essential for oocyte death under *Xist* OE overexpression, we calculated the number of oocytes in 0.5 dpp ovaries treated with 3MA. The number of oocytes that survived in the 3MA group was higher than that in the control group, while *Xist* overexpression partly abrogated this stimulatory effect of 3MA (Fig. 2E). Finally, the 0.5 dpp ovaries transfected with pcDNA-*Xist* were cultured for 96 h and then transplanted under the kidney capsule of ovariectomized adult recipient mice (8-wk old) and monitored for 14 d. Ovarian histology analysis revealed a significant loss of activated follicles in the *Xist*-overexpressed group compared to the controls (Fig. 2F, G). Together, these results suggested that *Xist* promotes perinatal oocyte loss through oocyte autophagy.

### *Xist* modulates *miR-23b-3p* and *miR-29a-3p* maturationin perinatal mouse ovaries

**Fig. 3:**
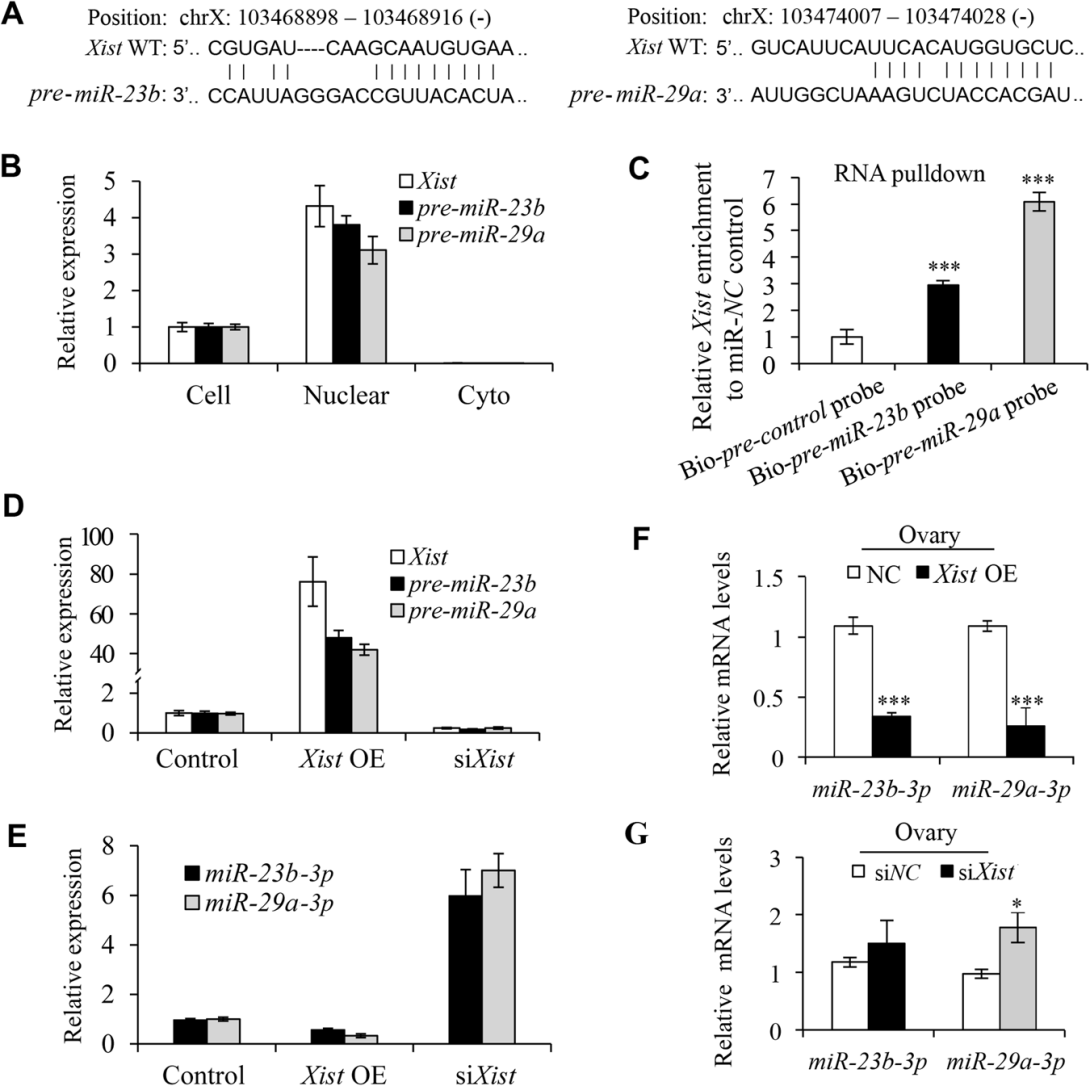
*Xist* regulates *miR-23b* and *miR-29a* maturation process. **a** The predicted *pre-miR-23b* and *pre-miR-29a* binding sites in the *Xist* transcript. **b** qRT-PCR analysis of *Xist, pre-miR-23b, and pre-miR-29a* expression in nuclear and cytoplasmic fraction relative to the whole cells from 0.5 dpp ovaries. **c** Enrichment of *Xist* pulled down by biotin-*pre-miR-23b*, biotin-*pre-miR-29a*, or biotin-*pre-miR-NC* control in the nuclear fraction from 3T3 cells. **d** qRT-PCR analysis of *Xist, pre-miR-23b, and pre-miR-29a* expression in *Xist* overexpression or *Xist* knockdown 3T3 cells. **e** qRT-PCR analysis of *miR-23b-3p, and miR-29a-3p* expression in *Xist* overexpression or *Xist* knockdown 3T3 cells. **f**, **g** Relative levels of *miR-23b-3p* and *miR-29a-3p* in cultured newborn ovaries transfected with *Xist* or empty control (**f**), and in ovaries transfected with si*Xist* or scrambled control (**g**). Student’s *t*-test: *P < 0.05, ***P < 0.001.

We next followed upon the underlying mechanism of *Xist* in regulating oocyte autophagy during perinatal PF formation. Recent studies have demonstrated that nuclear lncRNA regulates miRNA expression by suppressing its maturation process in the nucleus^33,34^. Given that *Xist* is also a nuclear lncRNA and that miRNAs are essential to promote oocyte survival and decrease apoptosis^26–29^, we wondered whether *Xist* may also play such a role in regulating oocyte death during PF formation. Previous studies have profiled differentially expressed miRNAs for association with premature ovarian failure development in both animal models and patients, including *miR-23b-3p* and *miR-29a-3p*^35,36^. We particularly selected these two miRNAs for further validation since the RNA fragments of the *pre*-*miR-23b* and *pre*-*miR-29a* are part of the *Xist* sequences (Fig. 3A), implying that *Xist* may regulate *miR-23b-3p* and *miR-29a-3p* expression. Additionally, accumulating studies have demonstrated that both *miR-23b-3p* and *miR-29a-3p* can regulate autophagy in a wide range of cells^37–39^, which may help raise the possibility that expression of *miR-23b-3p* and *miR-29a-3p* modulated by *Xist* in perinatal mouse ovaries is also associated with perinatal oocyte autophagy. To test this, we first confirmed that *pre*-*miR-23b* and *pre*-*miR-29a* were enriched in the nuclear fraction of cells from 0.5 dpp ovaries (Fig. 3B and Supplementary Fig. S1). Then, we applied a biotin-avidin pulldown assay to further delineate the direct interaction between *Xist* and *pre-miR-23b* and *pre*-*miR-29a* in the nucleus. Briefly, a biotin-labeled-specific anti-*pre-miR-23b* or anti-*pre*-*miR-29a* probe was synthesized and incubated with nuclear lysate from 3T3 cells. *Pre-miR-23b* or *pre*-*miR-29a* was co-precipitated via avidin-conjugated agarose beads, and the levels of *Xist* in the pulldown complex were analyzed by qRT-PCR. As shown in Fig. 3C, *Xist* was significantly enriched in the biotin-labeled *pre-miR-23b* or *pre*-*miR-29a* pulldown product compared to Bio-*pre*-*NC* control probe, suggesting that *Xist* can directly bind to *pre-miR-23b* or *pre-miR-29a* in the nucleus, thus preventing the export of *pre-miR-23b*/*pre*-*miR-29a* to the cytoplasm. Therefore, when we modulated *Xist* expression in 3T3 cells, we observed that both *pre*-*miR-23b* and *pre*-*miR-29a* levels were elevated upon *Xist* overexpression, and decreased upon *Xist* knockdown, compared to their respective controls (Fig. 3D). Correspondingly, overexpression of *Xist* decreased while depletion of *Xist* increased the expression of mature *miR-23b-3p* and *miR-29a-3p* (Fig. 3E). The expression of *Xist* positively correlated with the expression of *pre-miR-23b* and *pre*-*miR-29a*, but negatively with mature *miR-23b-3p and miR-29a-3p*, suggesting that *Xist* may regulate *miR-23b* and *miR-29a* maturation process.

To assess this hypothesis in vivo, we transfected 0.5 dpp mouse ovaries with pcDNA3.1-*Xist* or si*Xist*, and found that overexpression of *Xist* significantly increased the expression of *pre*-*miR-23b* and *pre*-*miR-29a*, while knockdown of *Xist* obviously decreased the expression of *pre*-*miR-23b* and *pre*-*miR-29a* in the nuclei (Supplementary Fig. S2). Correspondingly, both *miR-23b-3p* and *miR-29a-3p* levels were significantly decreased upon *Xist* overexpression (Fig. 3F), and upregulated upon *Xist* knockdown, compared to their respective controls (Fig. 3G). These results indicated that *Xist* may negatively regulate *miR-23b-3p*/*miR-29a-3p* expression in the neonatal mouse ovaries. Together, these data demonstrate that the maturation of *miR-23b-3p* and *miR-29a-3p* is associated with the expression of *Xist* in perinatal ovaries.

### *MiR-23b-3p*/*miR-29a-3p* are involved in the regulation of PF formation in the perinatal mouse ovaries

**Fig. 4:**
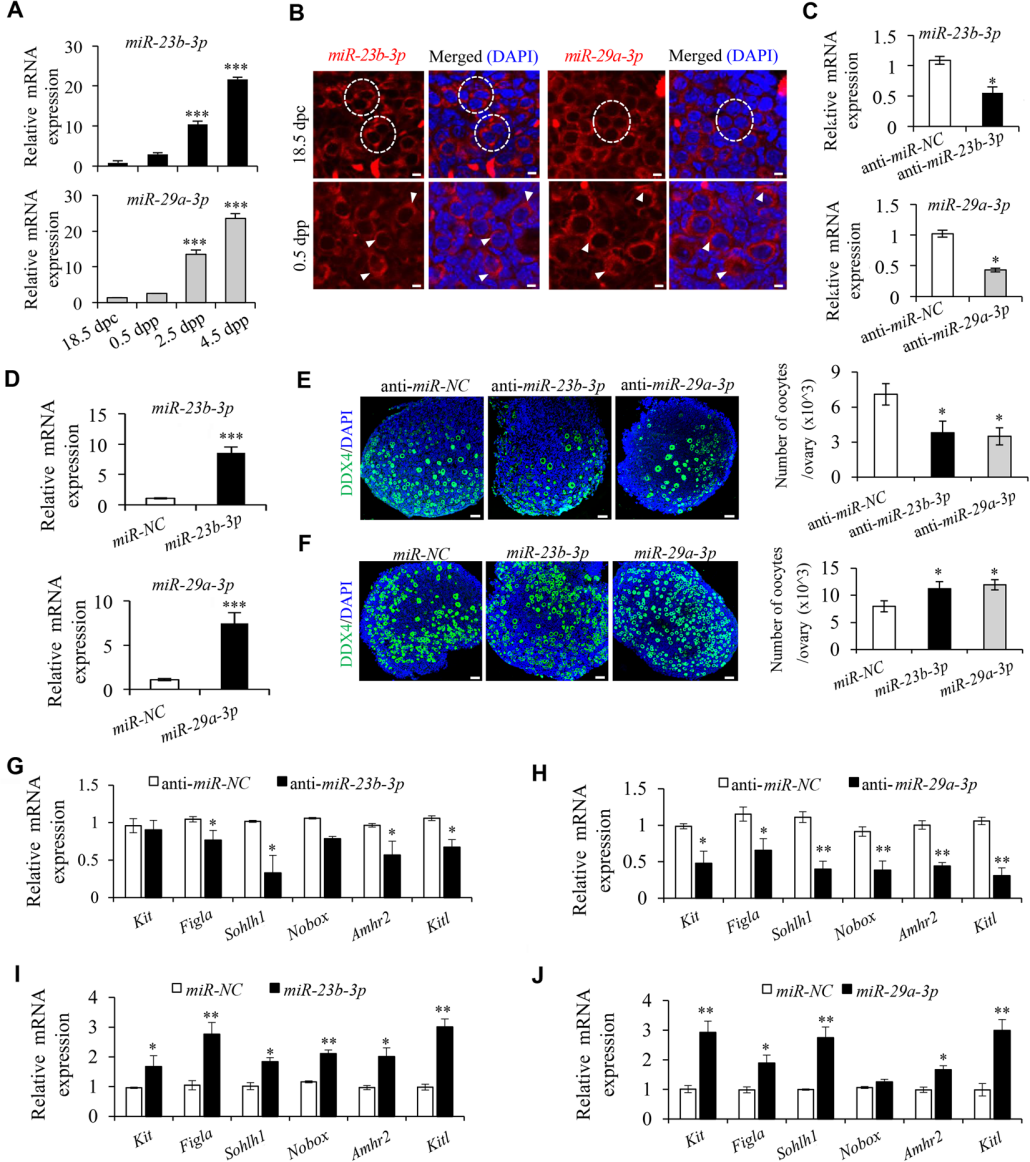
Expression of *miR-23b-3p* and *miR-29a-3p* are essential for PF formation in the perinatal mouse ovaries. **a** qRT-PCR analysis of *miR-23b-3p* (top) and *miR-29a-3p* (bottom) expression at the indicated time points. *Gapdh* was used to normalize samples. **b** RNA-FISH analysis of *miR-23b-3p* and *miR-29a-3p* expression in perinatal mouse ovaries from 18.5 dpc, and 0.5 dpp. The nucleus was stained by DAPI (blue). Dashed circle indicating representative cysts. Arrows heads indicating oocytes. Scale bars: 5 μm. **c**, **d** qRT-PCR analysis of *miR-23b-3p* (top) and *miR-29a-3p* (bottom) expression in newborn ovaries transfected with anti-*miR-23b-3p*, anti-*miR-29a-3p*, or anti-*miR-NC* (**c**), and mimics for *miR-23b-3p*, *miR-29a-3p*, *or miR-NC* (**d**). **e**, **f** Representative DDX4 immunofluorescence images (left) and quantification of follicles (right) in newborn ovaries transfected with anti-*miR-23b-3p*, anti-*miR-29a-3p*, or anti-*miR-NC* (**e**), and mimics for *miR-23b-3p*, *miR-29a-3p*, *or miR-NC* (**f**). Scale bars: 50 μm. **g**–**j** qRT-PCR analysis of genes involved in PF survival and development in newborn ovaries transfected with anti-*miR-23b-3p* (**g**), anti-*miR-29a-3p* (**h**), or anti-*miR-NC*, and mimics for *miR-23b-3p* (**i**), *miR-29a-3p* (**j**), *or miR-NC*. Student’s *t*-test: *P < 0.05, **P < 0.01, ***P < 0.001.

Given *Xist* negatively regulates mature *miR-23b-3p*/*miR-29a-3p*, we speculated to observe an inversed expression pattern of *miR-23b-3p*/*miR-29a-3p* in perinatal mouse ovaries to that of *Xist*. Actually, qRT-PCR analysis just confirmed that *miR-23b-3p*/*miR-29a-3p* expression increased significantly along with the establishment of the PF pool from 18.5 dpc to 4.5 dpp (Fig. 4A). In addition, the cytoplasmic localization of the *miR-23b-3p*/*miR-29a-3p* by RNA-FISH analysis in oocytes from both cysts and PFs can be readily detected (Fig. 4B). These data raised a possibility that the promoting effect of *Xist* on oocyte loss during PF formation is likely mediated by its negative regulation on *miR-23b-3p*/*miR-29a-3p* expression. To further assess the effect of *miR-23b-3p*/*miR-29a-3p* in PF formation, we silenced or overexpressed them in 0.5 dpp ovaries, respectively. The silencing or overexpression efficiency was then confirmed 96 h post transfection (Fig. 4C, D). Then, the cultured ovaries were subjected to histological examination. Immunohistochemistry analysis with anti-DDX4 antibody showed that anti-*miR-23b-3p* or anti-*miR-29a-3p* significantly decreased oocyte number compared to the controls (Fig. 4E), and overexpression of *miR-23b-3p* or *miR-29a-3p* exhibited more oocytes than the miRNA controls 96 h post transfection (Fig. 4F). Analysis of LC3B expression confirmed that inhibiting *miR-23b-3p* or *miR-29a-3p* in the newborn ovaries obviously elevated, while overexpression of *miR-23b-3p* or *miR-29a-3p* decreased oocyte autophagy compared to their respective controls (Supplementary Fig. S3). Furthermore, expression of the genes related to PF formation decreased in anti-*miR-23b-3p* or anti-*miR-29a-3p* treated ovaries (Fig. 4G, H), while increased in *miR-23b-3p* or *miR-29a-3p* overexpressed group (Fig. 4I, J). These results indicated that *miR-23b-3p*/*miR-29a-3p* have the opposite effect to that of *Xist*, which may promote PF formation in perinatal mouse ovaries, possibly by preventing oocyte loss.

### *Xist* relieves the inhibition of *miR-23b-3p*/*miR-29a-3p* on their common target STX17

**Fig. 5:**
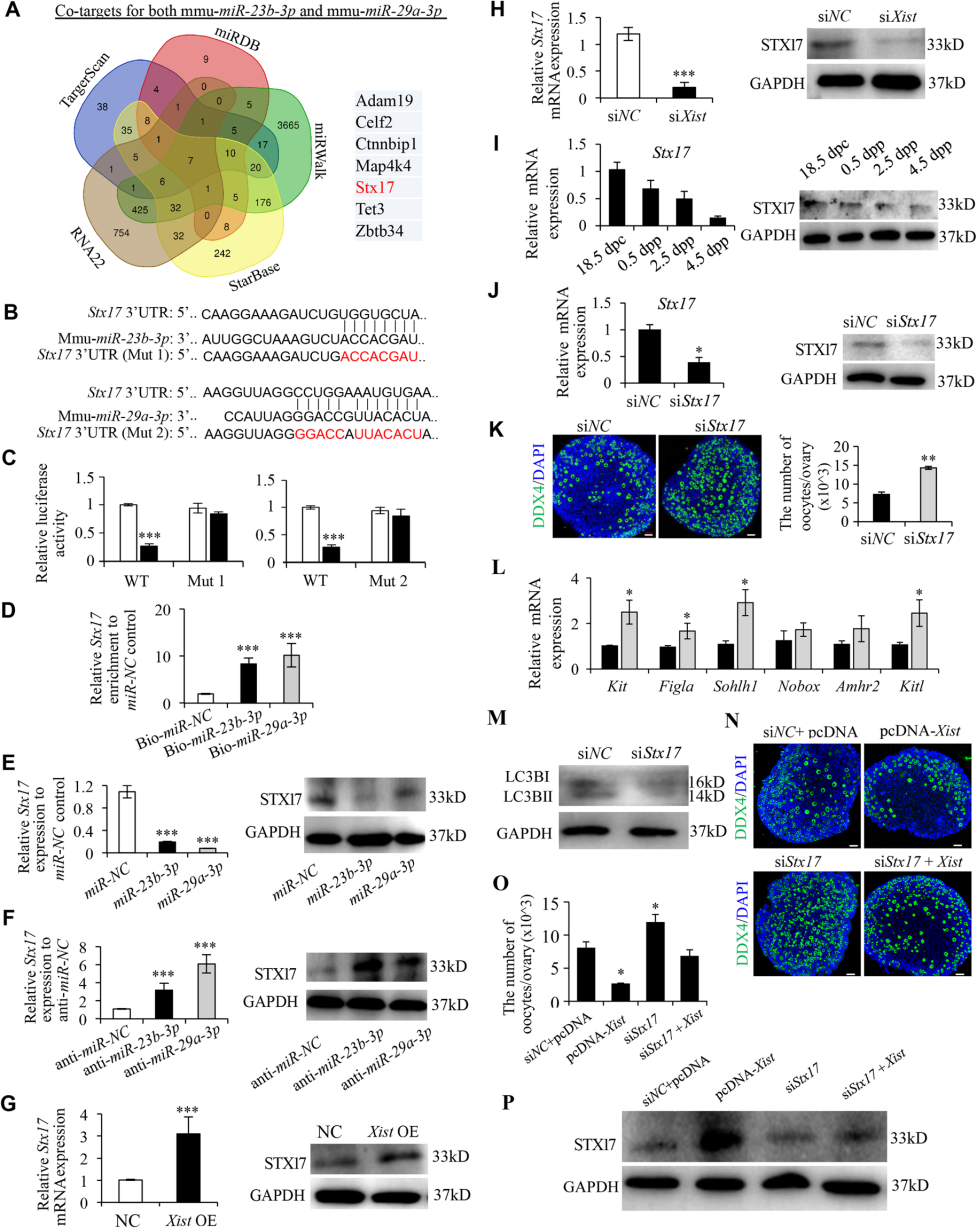
STX17 is a downstream target of *Xist*-*miR-23b-3p*/*miR-29a-3p* axis and regulates PF formation. **a** The Venn diagram shows overlapping of the co-target genes of *miR-23b-3p*/*miR-29a-3p* from five representative databases. **b** The predicted binding sites of *miR-23b-3p* and *miR-29a-3p* binding sites in the 3′-UTR of *Stx17*. The red nucleotides represent mutant sequences of target sites. **c** Luciferase activities in HEK293 cells transfected with WT or mutant *Stx17* plasmid together with *miR-23b-3p* or *miR-29a-3p*, or *miR-NC*. **d** Enrichment of *Stx17* pulled down by biotin-*miR-23b-3p*, biotin-*miR-29a-3*, or biotin-*miR-NC* control in 3T3 cells. **e**–**h** Relative *Stx17* mRNA (left), and STX17 protein (right) expression in cultured newborn ovaries transfected with mimics for *miR-23b-3p*, *miR-29a-3p*, *or miR-NC* (**e**), anti-*miR-23b-3p*, anti-*miR-29a-3p*, or anti-*miR-NC* (**f**), *Xist* or empty control (**g**), and si*Xist* or scrambled control (**h**). **i** Relative *Stx17* mRNA (left), and STX17 protein (right) expression at the indicated time points. **j** Relative *Stx17* mRNA (left), and STX17 protein (right) expression in cultured newborn ovaries transfected with si*Stx17*, or si*NC* as a control. **k** Representative images (left) and quantification of follicles (right) in newborn ovaries transfected with si*Stx17*, or si*NC*. Scale bars: 50 μm. **l** qRT-PCR analysis of genes involved in PF survival and development in newborn ovaries transfected with si*Stx17*, and si*NC* as a control. **m** WB analysis of LC3B expression in newborn ovaries treated under indicated condition. **n** Representative images of DDX4 in newborn ovaries co-transfected with pcDNA-*Xist* or empty vector together with *siStx17* or scrambled control. Scale bars: 50 μm. **o** Quantification of follicles in **n**. **p** WB analysis of STX17 expression in newborn ovaries treated under indicated condition. GAPDH was used as a loading control. Student’s *t*-test: *P < 0.05, **P < 0.01, ***P < 0.001.

To find out downstream genes sharing the regulatory role of *miR-23b-3p*/*miR-29a-3p* with *Xist*, we used five online bioinformatic tools (TargetScan, miRDB, miRWalk, Starbase, and RNA22) to predict the potential co-target genes of *miR-23b-3p*/*miR-29a-3p* (Fig. 5A). Among the seven co-target genes with sequence complementarity to both *miR-23b-3p*/*miR-29a-3p*, STX17 protein has attracted our particular attention, since it is directly implicated in the autophagy process^40^. Syntaxin 17 (STX17) is a soluble N-ethylmaleimide-sensitive factor attachment protein receptor (SNARE) protein that drives the maturation of autophagosomes acquiring degradative enzymes by fusing with the lysosome^41,42^. STX17 localizes to the outer membrane of autophagosomes, and binds directly to the autophagosomal protein, LC3^43^. Failure of autophagosome-lysosome binding leads to the blockade of the autophagic flux and abnormal degradation of autophagosomes^44^. Therefore, STX17 may function as the downstream target for *miR-23b-3p*/*miR-29a-3p* in regulating oocyte autophagy in the perinatal ovaries, and thus was selected for further analysis. We first synthesized wild-type (WT) and mutant *Stx17* 3′UTR (Mut1 corresponding to *miR-23b-3p*, and Mut2 to *miR-29a-3p*), and constructed them into luciferase reporters, separately (Fig. 5B). Luciferase reporter assays demonstrated that overexpression of *miR-23b-3p* or *miR-29a-3p* repressed luciferase activity in HEK293 cells transfected with WT *Stx17* 3′UTR reporter plasmid, whereas such effect was not observed in the mutant reporters (Fig. 5C). Next, biotin-labeled miRNA pulldown assays verified that *Stx17* is the target gene for *miR-23b-3p*/*miR-29a-3p* (Fig. 5D). Further analyses revealed that STX17 expression could be downregulated by overexpression of *miR-23b-3p* or *miR-29a-3p*, while upregulated by inhibitors for *miR-23b-3p* or *miR-29a-3p* (Fig. 5E, F). Since *Xist* inhibits the maturation of *miR-23b-3p*/*miR-29a-3p*, upregulation of *Xist* positively affected STX17 expression through modulating *miR-23b-3p*/*miR-29a-3p* (Fig. 5G), while an opposite effect was observed upon *Xist* inhibition (Fig. 5H). Together, these findings confirmed the existence of a *Xist*-*miR-23b-3p*/*miR-29a-3p*-STX17 regulatory axis in the perinatal ovaries.

### STX17 is responsible for *Xist*-mediated perinatal oocyte loss

To further verify STX17 is the downstream effector in *Xist*-*miR-23b-3p*/*miR-29a-3p*-STX17 regulatory axis in regulating perinatal oocyte loss, we first showed a similar expression pattern of STX17 to that of *Xist* in the perinatal mouse ovaries (Fig. 5I). Next, silencing STX17 in 0.5 dpp ovaries significantly increased the oocyte number compared to controls, after 96 h of in vitro culture (Fig. 5J, K). Additionally, expression of the genes related to PF formation also increased in si*Stx17*-treated ovaries (Fig. 5L). Furthermore, STX17-silenced neonatal ovaries showed a suppressed expression pattern of LC3B (Fig. 5M and Supplementary Fig. S4A). Conversely, we also overexpressed STX17 in the neonatal ovaries (Supplementary Fig. S4B), and observed that STX17 overexpression significantly decreased the oocyte number compared to controls after 96 h of in vitro culture (Supplementary Fig. S4C). Meanwhile, STX17-overexpressed neonatal ovaries showed an increased expression pattern of LC3B (Supplementary Fig. S4D). These data suggested that STX17 is involved in regulating PF pool formation through accelerating oocyte autophagy. Finally, to verify the ability of *Xist* to promote perinatal oocyte loss in an STX17-dependent manner, we treated 0.5 dpp ovaries with pcDNA3.1-*Xist*, si*Stx17*, or both in combination. The results demonstrated that knockdown of STX17 partially rescued the suppressive effects of *Xist* on PF pool formation (Fig. 5N–P).

### YY1 is essential for *Xist* transcription during PF pool formation

**Fig. 6:**
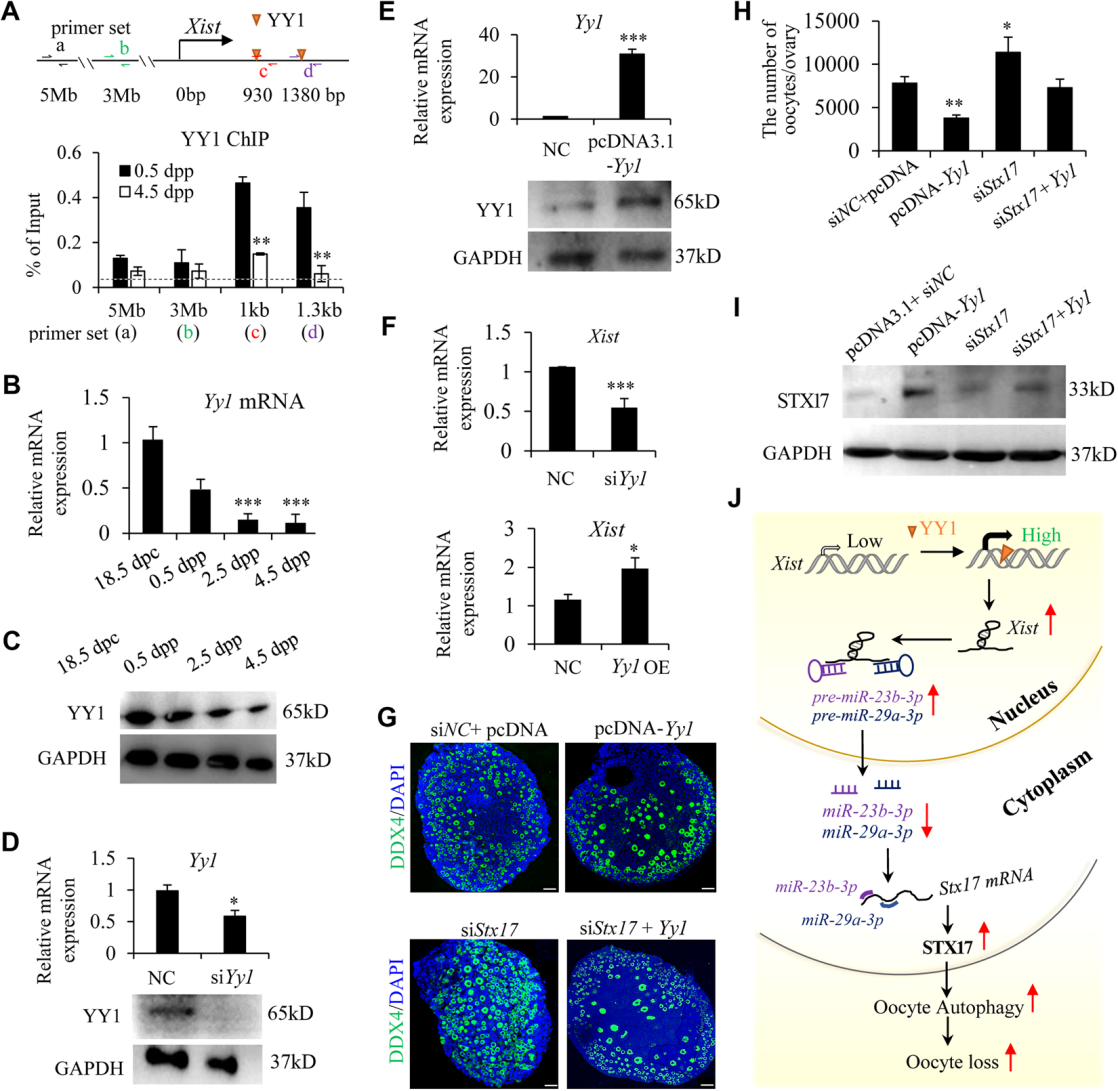
YY1 is essential for *Xist* expression and functions as an upstream regulator for *Xist*-*miR-23b-3p*/*miR-29a-3p-*Stx17 axis during PF formation. **a** Schematic representation for YY1 binding motif within the proximal promoter region of *Xist*, with respective primer sets to amplify YY1 binding region. The upstream control region is also represented (top). Bottom: ChIP analysis of YY1 binding in newborn mouse ovaries at 0.5 dpp, and 4.5 dpp. The dashed line represents the basal IgG binding. **b**, **c** Relative *Yy1* mRNA (**b**), and YY1 protein (**c**) expression in perinatal ovaries at indicted time. **d**, **e** Relative *Yy1* mRNA (top), and YY1 protein (bottom) expression in cultured newborn ovaries transfected with si*Yy1* or control (**d**), pcDNA3.1-*Yy1*, and NC control (**e**). **f** Relative *Xist* mRNA expression in cultured newborn ovaries transfected with si*Yy1* or siRNA control (top), pcDNA3.1-*Yy1* or NC control (bottom). **g**, **h** Representative DDX4 immunofluorescence images (**g**) and quantification of follicles (**h**) in newborn ovaries transfected under indicated condition. Scale bars: 50 μm. **i** WB analysis of STX17 expression in newborn ovaries treated under indicated condition. **j** Proposed model for *Xist*-*miR-23b-3p*/*miR-29a-3p-*STX17 axis in regulating oocyte loss in the perinatal mouse ovaries. Student’s *t*-test: *P < 0.05, **P < 0.01, ***P < 0.001.

It is essential for transcription factor Yin-Yang 1 (YY1) to bind directly to *Xist* promoter and activate *Xist* transcription in multiple cells^45,46^. To test whether YY1 facilitates *Xist* expression in the perinatal ovaries, we first performed ChIP assays using YY1 antibody and detected YY1 binding at *Xist* promoter with qPCR primers flanking the YY1 binding sites (Fig. 6A). We observed a strong enrichment of YY1 to this region in 0.5 dpp ovaries, while YY1 binding dramatically decreased in 4.5 dpp ovaries (Fig. 6A). This binding pattern was closely related to a rapid decrease of *Xist* expression from 18.5 dpc to 4.5 dpp ovaries, suggesting that YY1 binding may positively regulate *Xist* expression in the perinatal ovaries. Next, we examined the expression levels of YY1 during PF formation, and found a gradual decrease of YY1 expression in a time-specific manner from 18.5 dpc to 4.5 dpp (Fig. 6B, C). Given previous studies have shown that depletion of YY1 specifically inhibits *Xist* expression in other female cells^45,46^, our data raised a possibility that the decreased YY1 in perinatal ovaries may contribute to the downregulated *Xist*. To confirm this causal relationship between YY1 and *Xist* expression, gain or loss of function experiments were performed in 0.5 dpp ovaries by silencing or overexpressing YY1. The qRT-PCR and WB analyses showed that si*Yy1* transfection decreased, and pcDNA-*Yy1* transfection increased YY1 expression in 4.5 dpp ovaries (Fig. 6D, E). YY1 suppression significantly inhibited *Xist* expression, while overexpression of YY1 increased *Xist* expression (Fig. 6F). As a transactivator of *Xist*, YY1 was shown to play a similar role during PF pool formation to that of *Xist*, in an STX17-dependent manner (Fig. 6G–I). In agreement with the previous studies showing that YY1 is implicated in autophagy in multiple cells^47,48^, we then confirmed that YY1 positively regulated the LC3B protein expression in the newborn ovaries (Supplementary Fig. S5A). As we showed that *Xist* promotes perinatal oocyte loss through autophagy in an STX17-dependent manner (Figs. 2 and 5), YY1 overexpression also rescued the decreased LC3B expression mediated by STX17 depletion (Supplementary Fig. S5B). These data supported the existence of a YY1-*Xist*-*miR-23b-3p*/*miR-29a-3p*-STX17 regulatory axis during PF formation.

## Discussion

It is well accepted that cellular apoptosis and autophagy processes are responsible for programmed oocyte loss occurring briefly around the time of birth^5,31,40^. Meanwhile, lncRNAs are emerging as central regulators in controlling cell autophagy or apoptosis in various diseases^49,50^. However, there is no such lncRNA report in the process of PF formation. Here, we demonstrated that *Xist* promotes perinatal oocyte loss mainly through oocyte autophagy. Mechanically, we showed explicitly that, during the early period of PF pool formation, YY1 activates *Xist* transcription; *Xist* blocks *miR-23b-3a/miR-29a-3a* biogenesis process, resulting in decreased mature *miR-23b-3a/miR-29a-3a* expression; Given STX17 is a co-target for *miR-23b-3a/miR-29a-3a* and that STX17 is a critical factor in the process of autophagy, *Xist* relieving the inhibition of *miR-23b-3p*/*miR-29a-3p* on STX17 finally leads to an increased STX17 expression, which promotes massive oocyte loss through oocyte autophagy (Fig. 6J). These findings indicate that *Xist* is an indispensable primordial folliculogenesis factor that controls PF pool formation.

*Xist*, whose gene product is a lncRNA, is exclusively expressed on the inactivated X-chromosome and its abnormal expression has been linked strongly to the process of X-chromosome inactivation^51,52^. It is speculated that variation in expression of *Xist* gene that leads to a preferential silencing of genes on the X-chromosome related to the maintenance of ovarian function may consider being a susceptibility factor for POI^52^. Clinically, the inadequate number of follicles in the perinatally generated PF pool is one of the causative factors for POI, leading to the shortened reproductive life span. But whether *Xist* is involved in PF pool formation is unclear. The only available clue is coming from the transcriptome analysis of lncRNA expression in human primordial, primary and small antral follicles, with *Xist* being identified as one of highly expressed lncRNAs^15^. Our finding that interference of *Xist* expression affects the oocyte loss during the formation of PF pool may lend support to this hypothesis and help better understand the novel functional relationship between *Xist* and pathogenesis of POI.

Accumulating studies have demonstrated that, in addition to protein-mediated post-transcriptional control in miRNA biogenesis, nuclear ncRNAs can also function as negative regulators for miRNA biogenesis as miRNA maturation process begins in the nucleus. For example, colon cancer-associated transcript-2 (*Ccat2*), a lncRNA mainly located in the nucleus, has been reported to selectively block *miR-145* maturation by inhibiting *pre-miR-145* export to the cytoplasm in colon cancer cells^33^. Tang et al.^53^ reported that, in the cell nucleus, *miR-709* directly binds to *pri-miR-15a/16-1* and prevents its processing into *pre-miR-15a/16-1*, leading to a suppression of *miR-15a/16-1* biogenesis. In line with these reports, our findings that expression of *Xist* in the perinatal mouse ovaries positively correlated with expression of *pre*-*miR-23b/pre-miR-29a*, but inversely correlated with mature *miR-23b-3a/miR-29a-3a*, and that *Xist* binds to the *pre-miR-23b/pre-miR-29a* in the nucleus, imply that *Xist* may block the export of *pre-miR-23b/pre-miR-29a* to the cytoplasm, resulting in decreased mature *miR-23b-3a/miR-29a-3*. In the process of miRNA biogenesis, exportin-5^54^, and exportin-1^55^ are identified to be nuclear export factors to transport pre-miRNAs from the nucleus to the cytoplasm. It is not clear whether *Xist* affects the function of either exportin-5 or exportin-1 and this requires further investigation. Additionally, recent studies found that *Xist* functions as a competing endogenous RNA (ceRNA) to sponge the common miRNAs, and therefore insulates the miRNAs and facilitates the corresponding miRNA-targeted transcripts in tumorigenesis^56,57^, or in the regulation of inflammation and apoptosis^58^. Together, these studies significantly expand the biological function of *Xist*/miRNA/mRNA axis in regulating a broad spectrum of biological processes.

Transcriptional regulation of *Xist* has been widely investigated and several transcription factors are identified to be required for its transcription^51^. A recent study has carried out in silico analysis of *Xist* promoter regions in seven species of eutherian mammals and revealed clustered YY1 consensus binding sites evolutionarily well conserved in all species tested, including human and mouse. Furthermore, YY1 can directly bind to the *Xist* promoter region and promote *Xist* expression during initiation and maintenance of X-chromosome inactivation, suggesting that YY1 binding triggers the activity of the *Xist* promoter^46^. In consistence with this activity of YY1, our study in the perinatal ovaries also verified the binding of YY1 at *Xist* proximal promoter region, which is necessary for *Xist* transactivation during PF pool formation. Additionally, our findings that the same expression pattern of YY1 as that of *Xist* was observed in the perinatal ovaries, and that modulation of YY1 expression induced a similar phenotype during PF formation as that of *Xist*, strongly suggest that YY1 may function as an important upstream regulator in *Xist-miR-23b-3p*/*miR-29a-3p* axis.

Collectively, we elucidate a novel mechanism by which lncRNA *Xist*, activated by transcription factor YY1, blocks *miR-23b-3p*/*miR-29a-3p* maturation process by inhibiting *pre-miR-23b/pre-miR-29a* export to the cytoplasm, and relieves the inhibition of *miR-23b-3p*/*miR-29a-3p* on STX17, resulting in massive oocyte loss through oocyte autophagy in the perinatal ovaries. Our work may offer new insights into the mysteries of early folliculogenesis in mammalian ovaries, and highlight the potential clinical value of *Xist-miR-23b-3p*/*miR-29a-3p-*STX17 axis in the diagnosis and treatment of POI.

## Materials and methods

### Mice and ovary culture

All wild-type mice were purchased from the Animal Core Facility of Nanjing Medical University. The Animal Care and Use Committee of Nanjing Medical University approved all the animal experiments. The mice were housed under a 12/12-h dark/light cycle with free access to food and water at 20–22 °C. Mice were mated using timed mating, and the presence of a vaginal plug was defined as 0.5 dpp. Embryonic ovaries were collected at 18.5 dpc, and neonatal ovaries were collected at 0.5, 2.5, and 4.5 dpp. For ovary culture, 4 ovaries were randomly selected and placed in a 24-well dish and cultured in DMEM/F-12 medium (Gibco, Life Technologies, NY, USA) supplemented with penicillin-streptomycin and ITS at 37 °C under 5% CO_2_. The *miR-23b-3p*/*miR-29a-3p* mimics, inhibitors, and their corresponding scramble controls were ordered from GenePharma (Shanghai, China). The *Xist*, and *Yy1* overexpression plasmids, siRNAs against *Xist*, and *Yy1* were from General Biosystems (Anhui, China). The oligonucleotide sequences were listed in Supplementary Table 1. For ovaried treated with 2.5 mM of 3MA (S2767, Selleck, USA), dimethylsulfoxide (DMSO) was used as a control.

### Cell culture

Human embryonic kidney (HEK) 293 cells and mouse NIH/3T3 cells were purchased from the Cell Resource Center of the Shanghai Institute for Biological Sciences (Shanghai, China). Both cell types cultured in Dulbecco’s Modified Eagle’s medium (DMEM; HyClone, USA) supplemented with 10% fetal bovine serum (FBS; Gibco, USA) and 1% penicillin/streptomycin (P/S; Thermo Fisher Scientific, Rockford, IL, USA) and were incubated in a humidified incubator at 37 °C with 5% CO_2_.

### Quantitative real-time PCR

RNA was extracted from mouse ovaries using TRIzol (#15596026, Invitrogen, MA, USA), followed by reverse transcription with First Strand cDNA Synthesis Kit (K1621, Thermo Scientific, MA, USA), and qRT-PCR analysis with AceQ qPCR SYBR Green Master Mix (Q141-02, Vazyme, Jiangsu, China). The primers are summarized in Supplementary Table 2. The relative fold change of gene expression was calculated using the relative standard curve method (2^−ΔΔCt^).

### Isolation of nuclear and cytoplasmic fractions

Cells were washed three times with cold PBS, followed by centrifugation at 500 rpm for 5 min at 4 °C. Cell pellets were then resuspended in 1× Hypotonic Buffer (20 mM Tris, pH 8.0; 10 mM NaCl, 3 mM MgCl_2_, and 10% NP-40), and incubated on ice for 15 min. After centrifugation at 3000 rpm for 10 min at 4 °C. the supernatant portion (cytoplasmic extract) was transferred into the precooled tube. The pellet is the crude nuclear fraction. Nuclear pellets were then washed twice with cold PBS.

### Western blotting

Ovaries were lysed with RIPA buffer (CW2333, CWBIO, Beijing, China) with 1× protease inhibitor (CW2200, CWBIO). The supernatant was collected and about 30 μg of denatured protein was separated on a 10% SDS-PAGE gel, and transferred to a nitrocellulose membrane. The blots were rinsed in TBS containing 0.5% Tween-20 and blocked with 5% nonfat dry milk. After incubation with the primary antibodies overnight at 4 °C, including anti-LC3B (#2775, Cell Signaling Technology, 1:1000), anti-STX17 (#31261, Cell Signaling Technology, 1:1000), anti-YY1 (#22156-1-AP, Proteintech, 1:1000), anti-Lamin B1 (#12586, Cell Signaling Technology, 1:1000), anti-GAPDH (ab8245, Abcam, 1:2000), and anti-FLAG (#14793, Cell Signaling Technology, 1:1000), the blots were then washed and incubated with corresponding peroxidase-conjugated secondary antibody for 1 h at room temperature. The signals were visualized using an Enhanced Chemiluminescence Detection Kit (#32106, Thermo Scientific) on a Bio-Rad gel imaging system.

### Luciferase reporter assay

The wild-type or mutant *Xist* fragment containing the predicted binding sites of *pre-miR-23b* or *pre-miR-29a* was cloned into the modified pGL3 luciferase reporter vector (gift from Dr. Chun Lu from Nanjing Medical University). Luciferase reporter plasmid and 40 nM of *pre-miR-23b*, *pre-miR-29a-3p*, or *pre-control* were co-transfected into HEK293 cells. The luciferase reporter assay system (E2920, Promega) was used to measure luciferase activity.

### RNA fluorescent in situ hybridization (RNA-FISH)

5′-FAM-labeled *miR-23b-3p* or *miR-29a-3p* probes were designed and synthesized by General Biosystems (Anhui, China). Hybridizations were carried out using FISH Kit from GenePharma (Shanghai, China). Briefly, the ovaries were fixed in 4% paraformaldehyde and processed for serial paraffin sectioning at 5 μm thickness. After permeabilization, sections were hybridized with specific probes, and 6-diamidino-2-phenylindole (DAPI) was used to stain nuclei. All fluorescence images were captured using Point Scanning Laser Confocal Microscope (ZEISS, Germany).

### Biotin-labeled miRNA pulldown assay

Briefly, the 3′-end biotinylated *pre-miR-23b-3p*, *pre-miR-29a-3p,* or *pre-miRNA* control probe (50 nM, General Biosystems, Anhui, China) was transfected into 3T3 cell, and then cell nuclear fraction was extracted 48 h post transfection. Dynabeads MyOne Streptavidin C1 kit (Invitrogen) was used to enrich the biotin-coupled RNA complex. The beads were then pelleted and washed to remove unbound materials. Beads-bound RNA was extracted with TRIzol reagent. The abundance of *Xist* in the isolated fractions was tested by qRT-PCR.

### Immunofluorescence

Sections were deparaffinized, rehydrated, followed by antigen retrieval by boiling the sections in 0.01 M citrate buffer, pH 6.0 for 15 min. Then the sections were blocked in 10% normal goat serum, and incubated with primary antibodies overnight at 4 °C, including anti-DDX4 (Ab13840, Abcam, 1:200), anti-DDX4(Ab27591, Abcam, 1:200), and anti-LC3B (#2775, Cell Signaling Technology, 1:100). After 5 washes with PBS, the sections were incubated with secondary antibodies for 1 h at room temperature. DAPI was used to stain nuclei.

### Chromatin immunoprecipitation (ChIP)

MAGNA ChIP kit (#17371RF, Millipore, USA) was used to perform the ChIP assay. Cross-linked chromatin from mouse ovaries was prepared and immunoprecipitations were followed with primary antibodies, including anti-Ago2 (#2897, Cell Signaling Technology, 4 μg/sample), anti-YY1 (#22156-1-AP, Proteintech, 4 μg/sample) or rabbit IgG (#12–370, Millipore, 4 μg/sample). The enriched chromatin DNA was quantified by qPCR. Primers used are listed in Supplementary Table 2.

### TUNEL assay

Oocyte apoptosis was measured by TUNEL staining with a TUNEL Apoptosis Detection Kit (FITC) (Yeasen, Shanghai, China) on ovary sections. Nuclei DNA was stained with DAPI. The images were photographed using a Carl Zeiss (Oberkochen, Germany) lens.

### Transmission electron microscopy (TEM)

Ovaries were fixed in 2.5% glutaraldehyde in 0.2 M PBS (pH = 7.2) overnight at 4 °C, and processed and wrapped in epoxypropane resin following standard TEM procedures.

### Histological evaluation of follicle numbers

Theo varies were fixed, embedded in paraffin, and serially sectioned at a thickness of 5 μm. After staining, follicles were counted in every fifth section. To avoid duplicate counts, the only oocyte with a visible nucleus was counted. Germ cells not surrounded by GCs were scored as unassembled cysts. Germ cells surrounded by GCs or a mixture of squamous and cuboidal somatic cells were scored as primordial follicles. The total number of oocytes in each ovary was calculated by multiplication by 5.

### Statistical analysis

All experiments were repeated at least three independent biological replicates and presented as the mean ± the standard error of the mean. Statistical analysis was performed using Student’s *t*-tests or one-way ANOVA to compare the difference. A value of *P* < 0.05 was considered statistically significant.

## Supplementary information

SUPPLEMENTAL MATERIAL
