# Supplementary material for: Long non-coding RNA Xist regulates oocyte loss via suppressing miR-23b-3p/miR-29a-3p maturation and upregulating STX17 in perinatal mouse ovaries
Source: Cell Death Dis. 2021 May 25;12(6):540. doi: 10.1038/s41419-021-03831-4 (PMC8149765; doi:10.1038/s41419-021-03831-4)
Supplement: Supplementary file 1 — SUPPLEMENTAL MATERIAL [file 41419_2021_3831_MOESM1_ESM.docx]

**Long noncoding RNA *Xist* regulates oocyte loss via suppressing *miR-23b-3p*/*miR-29a-3p* maturation and upregulating STX17 in perinatal mouse ovaries**

**Supplementary Figures**


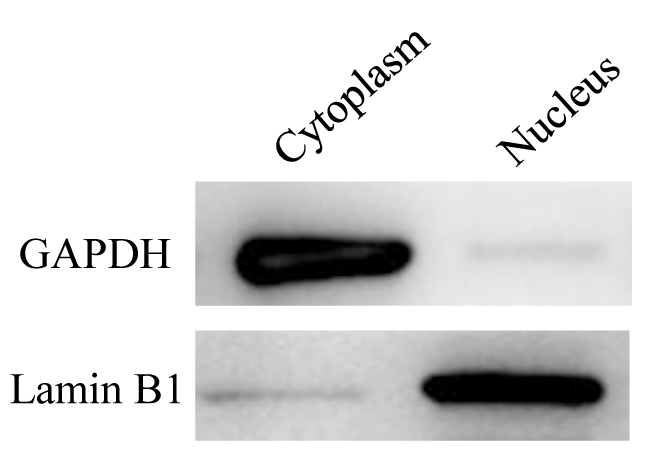


**Figure S1.** Cellular fractionation analysis of cells from 0.5 dpp ovaries, followed by Western blot with anti-GAPDH, and anti-Lamin B1 antibodies. Related to Figure 3.


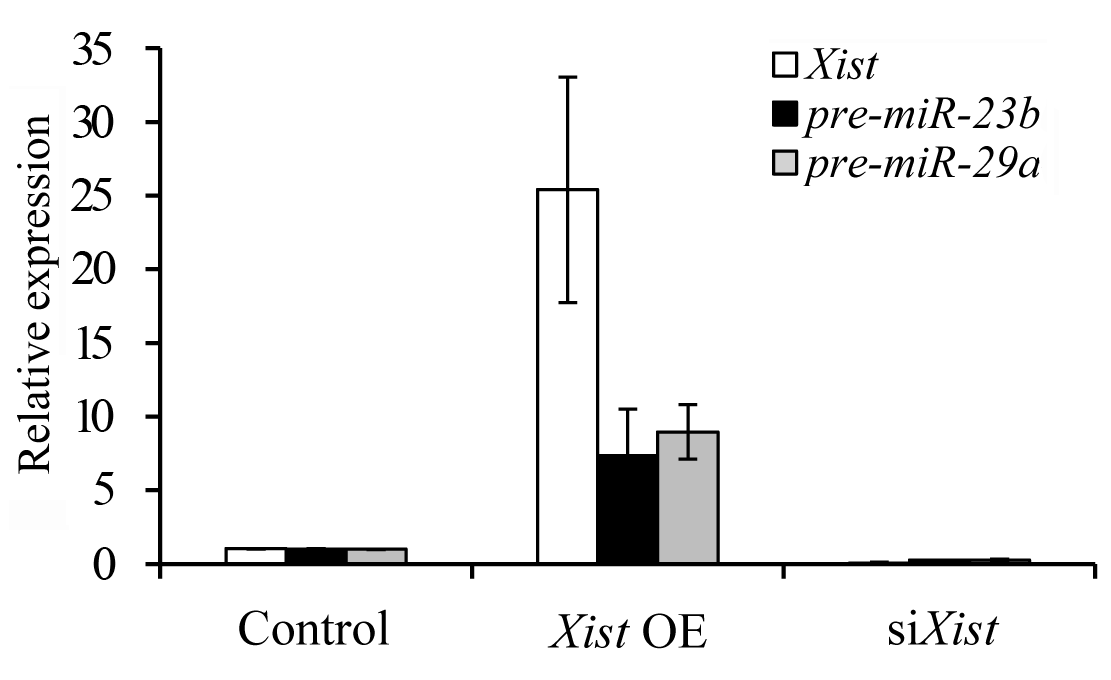


**Figure S2.** qRT-PCR analysis of *Xist, pre-miR-23b*, and *pre-miR-29a* expression in cultured newborn mouse ovaries transfected with pcDNA-*Xist* or si*Xist*. Related to Figure 3.


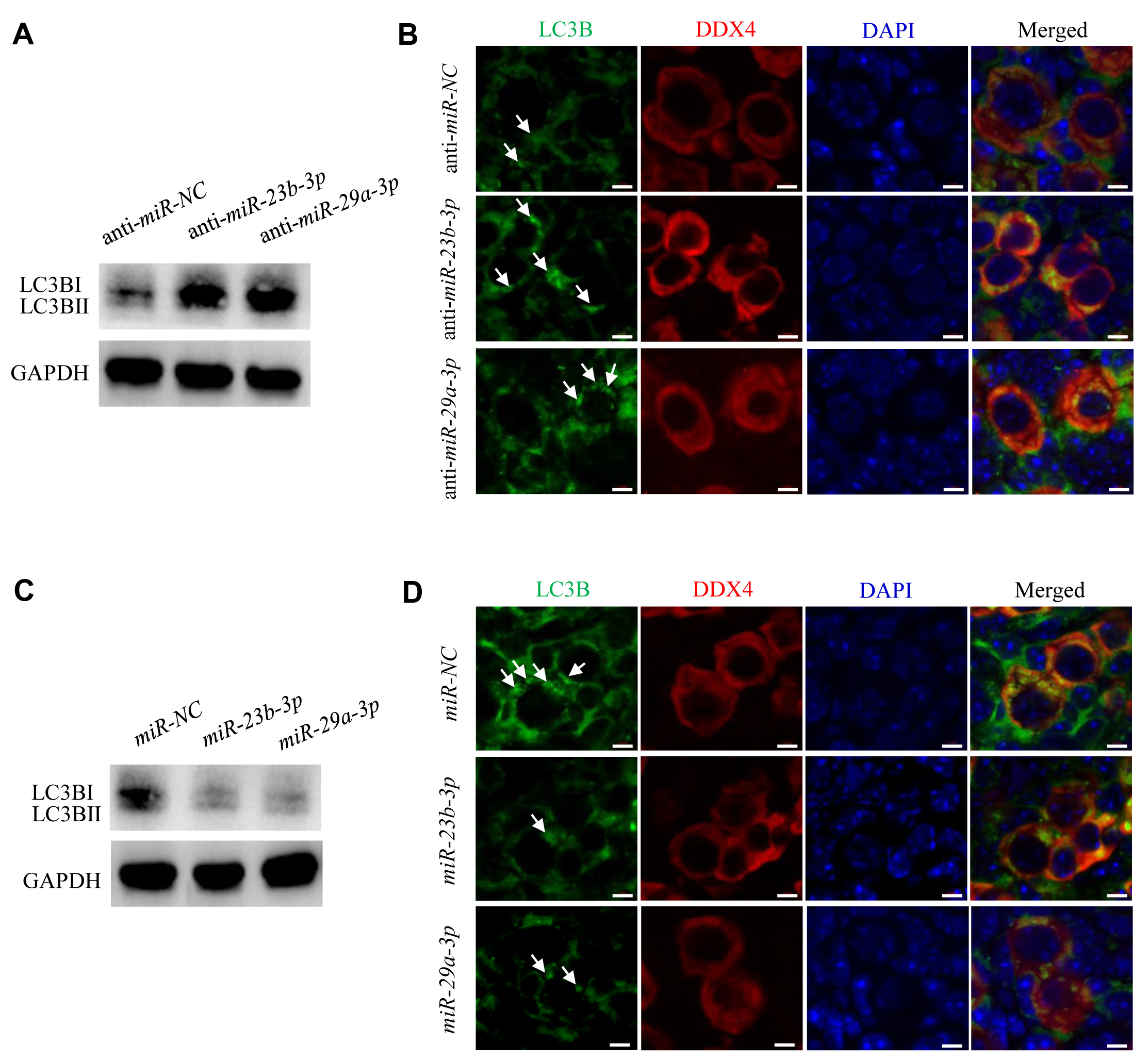


**Figure S3. a** WB analysis of active LC3B expression in newborn ovaries transfected with *anti-miR-23b-3p, anti-miR-29a-3p*, or *anti-miR-NC* with anti-LC3B antibody. GAPDH was used as loading control. **b** Immunofluorescence staining of LC3B (green), and DDX4 (red) in newborn ovaries transfected with *anti-miR-23b-3p, anti-miR-29a-3p, or anti-miR-NC*. The nucleus was stained by DAPI (blue). Scale bars: 5 μm. Arrows indicating LC3B puncta. **c** WB analysis of active LC3B expression in newborn ovaries transfected with mimics for *miR-23b-3p, miR-29a-3p*, or *miR-NC* with anti-LC3B antibody. GAPDH was used as loading control. **d** Immunofluorescence staining of LC3B (green), and DDX4 (red) in newborn ovaries transfected with mimics for *miR-23b-3p, miR-29a-3p*, or *miR-NC*. The nucleus was stained by DAPI (blue). Scale bars: 5 μm. Arrows indicating LC3B puncta. Related to Figure 4.


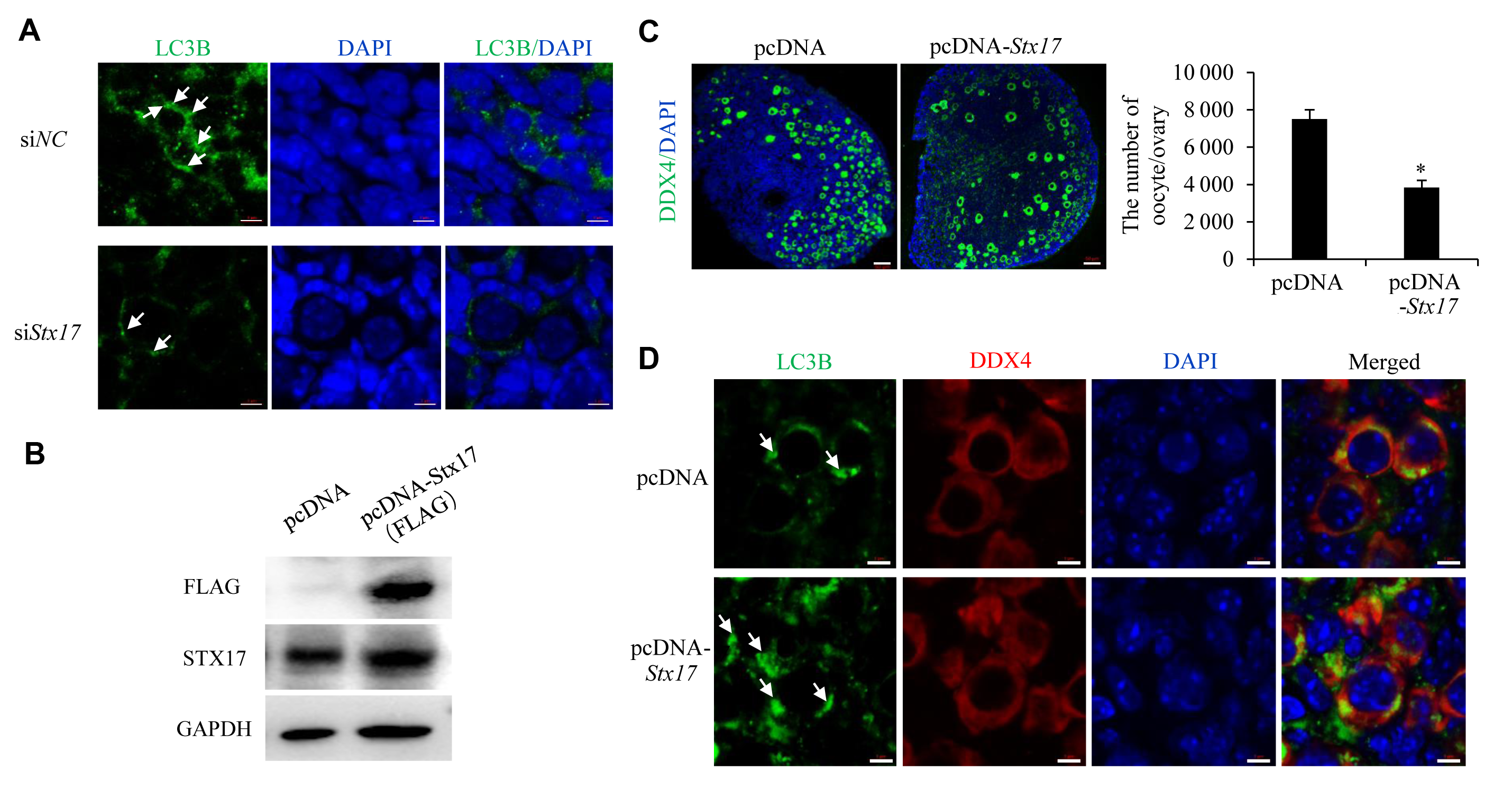
**Figure S4. a** Immunofluorescence staining of LC3B (green) in newborn ovaries transfected with si*Stx17*, and si*NC* as a control. The nucleus was stained by DAPI (blue). Scale bars: 5 μm. Arrow indicating LC3B puncta. **b** WB analysis of STX17 overexpreion with anti-FLAG, anti-STX17 antibodies in the newborn ovaries transfected with Flag-tagged pcDNA-*Stx17* or pcNDA3.1. GAPDH was used as loading control. **c** Representative images of DDX4 immunofluorescence staining (left) and quantification of follicles (right) in newborn ovaries transfected with pcDNA-*Stx17* or pcDNA3.1 empty control plasmid. Scale bars: 50 μm. **d** Immunofluorescence staining of LC3B (green), and DDX4 (red) in newborn ovaries transfected with pcDNA-*Stx17, or* pcNDA3.1. The nucleus was stained by DAPI (blue). Scale bars: 5 μm. Arrows indicating LC3B puncta. Related to Figure 5.


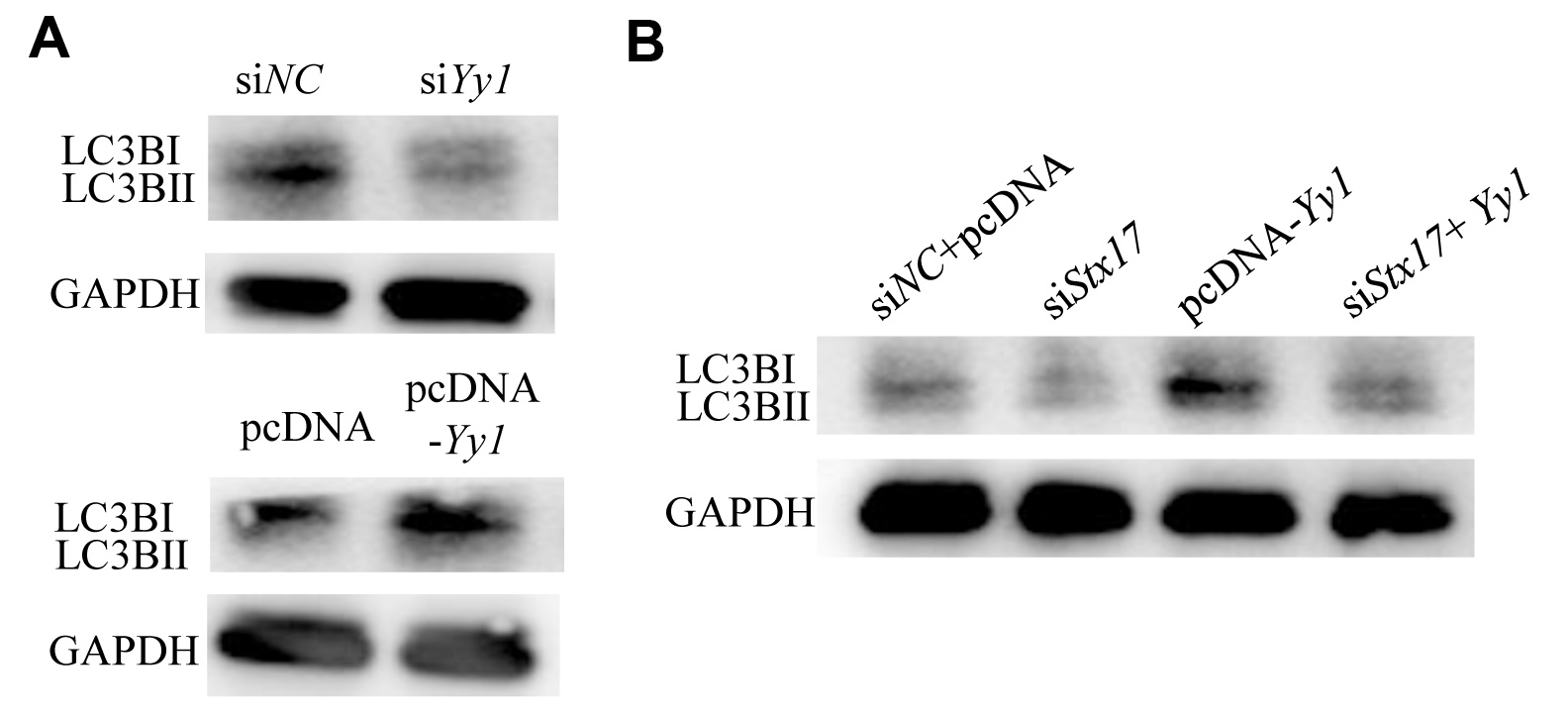


**Figure S5. a** WB analysis of LC3B expression in newborn ovaries transfected with si*Yy1* or si*NC* (top), pcDNA-*Yy1* or pcDNA control (bottom). GAPDH was used as a loading control. **b** WB analysis of LC3B expression in newborn ovaries treated under indicated condition. GAPDH was used as a loading control. Related to Figure 6.

**Supplementary Table S1. Oligonucleotide sets used in this study**

| **miRNA or siRNA** | **Sequences (5’-3’)** |
| --- | --- |
| *miR-NC* mimics | 5’-UUCUCCGAACGUGUCACGUTT  5’-ACGUGACACGUUCGGAGAATT |
| *miR-23b-3p* Mimics | 5’-UUCUUCGAACGUGUCACGUTT  5’-ACGUGACACGUUCGGAGAATT |
| *miR-29a-3p* Mimics | 5’-UAGCACCAUCUGAAAUCGGUUA  5’-ACCGAUUUCAGAUGGUGCUAUU |
| anti-*miR-NC* | 5’-CAGUACUUUUGUGUAGUACAA |
| anti-*miR-23b-3p* | 5’-GGUAAUCCCUGGCAAUGUGAU |
| anti-*miR-29a-3p* | 5’-UAACCGAUUUCAGAUGGUGCUA |
| si*NC* | 5’-UUCUCCGAACGUGUCACGUTT  5’-ACGUGACACGUUCGGAGAATT |
| si*Xist* | 5’-AUAACAGUAAGUCUGAUAGAGGACATT  5’-UGUCCUCUAUCAGACUUACUGUUAUTT |
| si*Pten* | 5’-AAUAUGCACAUAUCAUUACACTT  5’-GUGUAAUGAUAUGUGCAUAUUTT |
| si*Yy1* | 5’-AGAAGCAGGUGCAGAUCAATT  5’-UUGAUCUGCACCUGCUUCUTT |
| si*Stx17* | 5’-CCUUAGAAGCGGACUUAAUTT  5’-AUUAAGUCCGCUUCUAAGGTT |
| 5’FAM-*miR-NC* | 5’-UCUACUCUUUCUAGGAGGUUGUGA |
| 5’FAM*-miR-23b-3p* | 5’-AUCACAUUGCCAGGGAUUACC |
| 5’FAM*-miR-29a-3p* | 5’-UAGCACCAUCUGAAAUCGGUUA |
| Bio-anti-pre-*miR-23b* | 5'-ATCACATTGCCAGGGATTACC-3' |
| Bio-anti-pre-*miR-29a* | 5'-TAGCACCATCTGAAATCGGTTA-3' |
| Bio-anti-control probe | 5'-TGATGTCTAGCGCTT GGGCTTTG-3' |

**Supplementary Table S2. Primer sets used in this study**

| **Gene (mouse)** | **Primer sequences (5’-3’)** |
| --- | --- |
| *Figla* | F: 5’- ACAGAGCAGGAAGCCCAGTA |
|  | R: TGGGTAGCATTTCCCAAGAG |
| *Nobox* | F: 5’-CATGAAGGGGACCTGAAGAA |
|  | R: 5’-GGAAATCTCATGGCGTTTGT |
| *Sohlh1* | F: 5’-GGGCCAATGAGGATTACAGA |
|  | R: 5’-AAGTTTGCAGCAGCCACAG |
| *Kitl* | F: 5’-GAATCTCCGAAGAGGCCAGAA |
|  | R: 5’-GCTGCAACAGGGGGТAACAT |
| *Kit* | F: 5’-CTCCCCCAACAGTGTATTCAC |
|  | R: 5’-TAGCCCGAAATCGCAAATCTT |
| *Amhr2* | F: 5’-GCAGCACAAGTATCCCCAAAC |
|  | R: 5’-GTCTCGGCATCCTTGCATCTC |
| *Xist* | F: 5’-CCCGCTGCTGAGTGTTTGATA |
|  | R: 5’-CAGAGTAGCGAGGACTTGAAGAG |
| *Pten* | F: 5’-TGACCTCGCCACACTTGATA |
|  | R: 5’-AACCCACTGATAAGCCAACG |
| *Yy1* | F: 5’-AAAGCATCTGCACACCCACG |
|  | R: 5’-CTCCGGTATGGATTCGCACA |
| *Stx17* | F: 5’-GCTTGAGCCAGCGATACAGA |
|  | R: 5’-TATTGGAGCGCAGTTGCTGA |
| *Gapdh* | F: 5’-AGGTTGTCTCCTGCGACTTCA |
|  | R: 5’-GGGTGGTCCAGGGTTTCTTACT |
| *Xist*TSS 3M | F: 5’-GGAGAGATGGCCCTACAGGT |
|  | R: 5’-TTGTGTGCATTGCTGTGCTG |
| *Xist*TSS 5M | F: 5’-AGGAAGGAGTGGGATGTGGA |
|  | R: 5’-GTGGTGTGTGTTTCCATGCC |
| *Xist*TSS 1kb | F: 5’-ACGGCTATTCTCGAGCCAGTT |
|  | R: 5’-ATTGGTTGCTTTTATCCAGT |
| *Xist* TSS 1.3kb | F: 5’-GTCTATAAAATGGCGGCTCG |
|  | R: 5’-CGCCATCTTTTCCTGTACG |
| *miR-23b-3p* | F: 5’-ATCACATTGCCAGGGATTACC |
|  | R: 5’-GTCGTATCCAGTGCGTGTCGT |
| *miR-29a-3p* | F: 5’-TAGCACCATCTGAAATCGGTT |
|  | R: 5’-GTCGTATCCAGTGCGTGTCGT |
| *U6* | F: 5’-CTCGCTTCGGCAGCACATATACT |
|  | R: 5’-ACGCTTCACGAATTTGCGTGTC |
